# Sequence-independent RNA sensing and DNA targeting by a split domain CRISPR–Cas12a gRNA switch

**DOI:** 10.1093/nar/gkab100

**Published:** 2021-02-22

**Authors:** Scott P Collins, William Rostain, Chunyu Liao, Chase L Beisel

**Affiliations:** Department of Chemical and Biomolecular Engineering, North Carolina State University, Raleigh, NC 27695, USA; Génomique Métabolique, Genoscope, Institut François Jacob, CEA, CNRS, Univ Evry, Université Paris-Saclay, 91057 Évry, France; Helmholtz Institute for RNA-based Infection Research (HIRI)/Helmholtz Centre for Infection Research (HZI), Josef-Schneider-Str. 2/D15, 97080 Würzburg, Germany; Department of Chemical and Biomolecular Engineering, North Carolina State University, Raleigh, NC 27695, USA; Helmholtz Institute for RNA-based Infection Research (HIRI)/Helmholtz Centre for Infection Research (HZI), Josef-Schneider-Str. 2/D15, 97080 Würzburg, Germany; Medical Faculty, University of Würzburg, 97080 Würzburg, Germany

## Abstract

CRISPR technologies increasingly require spatiotemporal and dosage control of nuclease activity. One promising strategy involves linking nuclease activity to a cell's transcriptional state by engineering guide RNAs (gRNAs) to function only after complexing with a ‘trigger’ RNA. However, standard gRNA switch designs do not allow independent selection of trigger and guide sequences, limiting gRNA switch application. Here, we demonstrate the modular design of Cas12a gRNA switches that decouples selection of these sequences. The 5′ end of the Cas12a gRNA is fused to two distinct and non-overlapping domains: one base pairs with the gRNA repeat, blocking formation of a hairpin required for Cas12a recognition; the other hybridizes to the RNA trigger, stimulating refolding of the gRNA repeat and subsequent gRNA-dependent Cas12a activity. Using a cell-free transcription-translation system and *Escherichia coli*, we show that designed gRNA switches can respond to different triggers and target different DNA sequences. Modulating the length and composition of the sensory domain altered gRNA switch performance. Finally, gRNA switches could be designed to sense endogenous RNAs expressed only under specific growth conditions, rendering Cas12a targeting activity dependent on cellular metabolism and stress. Our design framework thus further enables tethering of CRISPR activities to cellular states.

## INTRODUCTION

CRISPR technologies continue to expand in scope and application from their first demonstration as programmable genome-editing tools 7 years ago (1–3). These technologies rely on programmable CRISPR-associated (Cas) nucleases directed by guide RNAs complementary to protospacer-adjacent motif (PAM)-flanked nucleic-acid targets, driving cleavage and repair. However, more recent work has advanced genome editing to treat specific genetic diseases, spread genes through wild insect populations, combat infectious diseases, detect biomarkers at the point-of-care, modulate the epigenetic state of a cell, and allow live imaging of cellular components (4–7). Early efforts to improve CRISPR technologies focused on engineering the nuclease for expanded site recognition and reduced off-target editing (8,9). However, as CRISPR technologies have begun to expand into applications such as germline editing and gene regulation, greater control over the timing, localization and strength of CRISPR activity becomes necessary. Excess levels of certain CRISPR components have been associated with increased off-target editing in eukaryotic cells and cytotoxicity in bacteria (10–13). For genome editing in the clinic, safety concerns could be partially alleviated by limiting editing activity to specific cell types or tissues linked to the genetic disease. For CRISPR-based gene drives, precise timing could help ensure viable offspring, while limiting nuclease activity to germline cells could reduce fitness defects. Finally, programmable CRISPR-based transcription factors could be better utilized when their functionality is tied to a wider regulatory network.

To address this growing need, multiple strategies have been recently reported to regulate the expression, activity and stability of different CRISPR components. Exogenous control over nuclease activity has been achieved using light, temperature and the presence of a small molecule (14–16). Alternatively, CRISPR activity can be regulated by endogenous intracellular factors, enabling autonomous control. The means to link CRISPR activity and intracellular states has been established by employing regulated promoters, fused aptamers, or by requiring cleavage by a tissue-specific microRNA (17,18). However, a growing set of approaches build on concepts in RNA engineering and, in particular, on toehold-mediated strand displacement (19–22). For these approaches, a designed RNA switch adopts a conformation that leaves an unpaired and highly accessible RNA sequence called the toehold. The toehold hybridizes to a separate ‘trigger’ RNA, facilitating further base pairing to drive a conformational and functional change in the toehold RNA switch. Toehold RNA switches have been widely adopted and applied to transcriptional and translational regulation, *in vitro* diagnostics, or biological computation (23–26). Combining toehold switches with gRNAs therefore could render the gRNA dependent on the presence or absence of virtually any RNA, tapping into the information-rich transcriptional profile of a cell to control CRISPR activity.

Existing gRNA switch designs have focused on two common Cas nucleases, Cas9 and Cas12a. Four groups engineered single-guide RNAs (sgRNAs) utilized by the Cas9 nuclease (19–21,27). The designs allowed the sgRNA to become functional or non-functional upon hybridization to a RNA trigger, where hybridization altered the accessibility of the DNA-targeting guide sequence and/or the conserved ‘Cas9 handle’ required for Cas9 binding. Separately, one group engineered gRNAs utilized by the Cas12a nuclease, which can process CRISPR arrays into multiple gRNAs without accessory factors and was shown to be more specific than Cas9 (22). Oesinghaus and Simmel engineered the Cas12a gRNA to become active upon hybridization to an RNA trigger, where hybridization unlocked the hairpin required for Cas12a recognition of the gRNA and displaced a hairpin sequestering the guide sequence. Recognition of the activated gRNA switch by Cas12a resulted in removal of the 5′ sensory domain by the RNAse activity of Cas12a, resulting in an otherwise ‘natural’ gRNA.

While these strategies demonstrated the potential of rendering guide RNAs dependent on cellular transcripts, the fundamental design schemes placed major sequence constraints on the RNA trigger or target DNA sequence. These constraints arose from one domain being dually responsible for competitively base pairing with the sensed RNA and with either the fixed Cas recognition sequence or the defined guide sequence within the gRNA. Competitive binding inherently couples two sequences, resulting in a design constraint. The study with Cas12a developed a workaround to this constraint by incorporating a second ‘bridging’ synthetic RNA, although this setup requires a slow-acting tripartite interaction for Cas12a activation and still contains some sequence constraints (22). Separately, the study with Cas9 by Jin and coworkers achieved sequence independence by controlling the secondary structure immediately upstream of the guide, although the extended 5′ end can interfere with Cas9 binding and activity, and this design scheme remains to be validated *in vivo* (19). Therefore, there remains an existing need for design schemes that uncouple the sequence of the sensed RNA from the gRNA.

Here, we demonstrate a distinct design scheme for Cas12a gRNAs that uncouples these sequences, allowing native transcripts and cellular signals to drive Cas12a activity *in vivo*. The modular design scheme involves a 5′ extension to the gRNA composed of a clamp and a split sensory domain further comprising a toehold and a loop. The clamp blocks formation of the hairpin required for Cas12a binding, while the sensory domain hybridizes to an RNA ‘trigger’ that drives an alternative conformation of the gRNA that can be bound and processed by Cas12a. Critically, the handle-blocking clamp acts independently of the sensory domain, allowing the RNA trigger and guide sequences to be selected independently. Using a design pipeline based on RNA-folding predictions coupled with cell-free transcription-translation systems (TXTL) and gene-repression in *Escherichia coli*, we show that designed gRNA switches could respond to different triggers and target different sequences orthogonally. The performance of the gRNA switches could be further modulated by altering the sequence and length of the clamp, toehold and loop, mediating the ‘rescue’ of an otherwise poorly functioning switch. Finally, we linked Cas12a activity in *E. coli* to different cellular states, including iron starvation by sensing the small RNA RyhB as well as L-arabinose utilization by sensing the mRNA encoding the catabolic enzyme AraB. Thus, sensory and targeting sequences can be decoupled when designing gRNA switches to leverage the transcriptional profile of a cell for autonomous control over CRISPR activity.

## MATERIALS AND METHODS

### Strains, plasmids and synthesized DNA

All strains, plasmids, oligos and gBlocks used in this work can be found in Supplementary Tables S1 and S2, including links to annotated sequences. All PCR amplifications were performed using the Q5 Hot Start High-Fidelity 2X Master Mix (NEB, Cat: M0494S). Plasmids for synthetic trigger testing were constructed using golden gate assembly. Reaction protocols and type IIS restriction enzyme overhangs generally followed the yeast toolkit (YTK) golden gate system (28).

To assemble plasmids for testing of synthetic RNA-triggered switches in *E. coli*, gRNA switch and trigger sequences were assembled with the constitutive sigma 70 promoter J23119 into a ColE1 high-copy backbone conferring kanamycin resistance by three-piece BsaI assembly. These transcriptional units were further cloned into a pBR332 low-copy backbone conferring ampicillin resistance by BsmBI assembly for use in *E. coli*.

For endogenous RNA sensing, gRNA switches were cloned from synthesized gBlock linear DNA into a low-copy pBR332 backbone conferring ampicillin resistance by two-piece BsaI assembly. gBlocks were synthesized to include terminal BsaI sites, while the pBR332 backbone included either a GFP or RFP dropout sequence with flanking BsaI sites. Plasmid-based expression of the araB or lacZ mRNA triggers were cloned into the same backbone as the dFnCas12a plasmid CB482 by gibson assembly (NEB, NEBuilder Hifi DNA Assembly Master Mix, E2621S) according to manufacturer's instructions. The *araB* gene, *lacZ* gene and pCB482 plasmid backbone were amplified for gibson assembly with SPCqpr1577–1582 and Q5 2X HotStart Master Mix. After Gibson assembly and clone verification, plasmid DNA was purified and the *E. coli* sigma 70 constitutive promoter J23119 was inserted upstream of the *araB* or *lacZ* genes by Q5 site-directed mutagenesis (NEB, Cat: 0554S) according to manufacturer's instructions to produce SPC1191 and SPC1192.

Synthesized linear DNA for use in cell-free TXTL reactions was ordered as gBlocks from IDT. gBlocks were resuspended in nuclease free water and directly used in TXTL reactions. If necessary, gBlock DNA was amplified using the Q5 Hot Start High-Fidelity 2X Master Mix (NEB, Cat: M0494S) with SPCpr044 & SPCpr045. PCR reactions were purified using a clean-and-concentrate kit (Zymo, Cat: D4013) and eluted in nuclease-free water.

### Golden gate reaction conditions

BsaI or BsmBI assembly reactions included approximately 20 fmol of each plasmid or amplicon assembly fragment in equimolar amounts, in addition to the following: 1 μl of T4 DNA Ligase buffer (NEB, Cat: B0202S), 0.5 μl BsaI or BsaI HFv2 (NEB, Cat:R3733S) or 0.5 μl BsmBI (NEB, Cat: R0580S), 0.5 μl of T7 DNA Ligase (NEB, Cat: M0318S) and nuclease-free water up to a final reaction volume of 10 μl. Reactions were incubated in thermocyclers with 20–35 cycles of (1: 5 min of digestion at 37°C for BsaI and 2 min at 42°C for BsmBI followed by 2: 5 min of ligation at 16°C) followed by a 30-min final digestion at 37°C or 42°C and a 10-min heat inactivation at 80°C.

### Growth conditions

All strains were cultured at 37°C. Cultures of 1 ml or 2 ml were grown in 14-ml falcon tubes shaking at 250 RPM. Cultures of 25 or 50 ml were grown in 125-ml or 250-ml shake flasks at 250 RPM. Larger-scale cultures were performed in Greiner Bio-One 96-deep-well plates (Greiner Bio-One Cat: 780271FD) covered with an adhesive gas-permeable membrane (Thermo Scientific Cat: AB0718). 750 μl of M9 minimal medium or LB medium was added to each well (see components below), shaking at 900 RPM in a Fisher incubating mini-shaker (FisherSci, Cat: 02-217-753). LB medium (10 g/l tryptone, 5 g/l yeast extract, 5 g/l NaCl) was used for synthetic and RyhB triggered gRNA testing, while M9 minimal medium (1 × M9 salts, 2 mM MgSO_4_, 0.1 mM CaCl_2_, 10 μg/ml thiamine hydrochloride) containing 0.2% casamino acids and 0.4% (w/v) glucose or arabinose was used for characterizing l-arabinose-responsive gRNA switches. All strains were plated on LB agar (LB medium with 1.5% agar) in 100 × 15 mm polystyrene petri dishes. To maintain any transformed plasmids, cells were cultured in liquid medium or on agar plates containing appropriate antibiotics at the following concentrations: 100 μg/ml of ampicillin, 34 μg/ml of chloramphenicol, 50 μg/ml of kanamycin.

### Flow cytometry analysis

Exponential-phase cultures were diluted 1:100 in 1× phosphate-buffered saline (1× PBS) and loaded into a flat-bottom 96-well plate (Olympus Cat: 25-104). The plate was loaded onto an Accuri C6 Flow Cytometer (Becton Dickinson) equipped with CFlow plate sampler, a 488-nm laser and a 530 ± 15 nm bandpass filter. Events were gated on forward scatter and side scatter. Thresholds of 14 000 for forward scatter and 600 for side scatter were used to reduce the measurement of non-cell particulates. The gate was set using *E. coli* cells stained with the DRAQ5 dye (Thermo Fisher Scientific). The fluorescence of the gated cells was then measured in FL1-H. At least 20 000 events surpassing thresholds were analyzed for each sample.

### Design of gRNA switches

Switch structural parameters for ON and OFF states were first defined and converted to DU+ notation (see Supplementary Table S3). For switches triggered by artificial sRNAs, the variable regions and the sRNA trigger were then generated using the NUPACK design function (29,30) using the structural constraints given in Supplementary Table S3, with the following parameters: temperature = 37°C, material = RNA, stop condition for switch OFF and ON states = 1% and 5%, respectively. Prevented patterns: AAAA, CCCC, GGGG, UUUU, KKKKKK, MMMMMM, RRRRRR, SSSSSS, WWWWWW, YYYYYY. For RyhB and *araB* mRNA-triggered switches, subsections of the RNAs were picked, and switch toehold and loops were designed to be complementary to these subsections. The *araB* CDS RNA was first folded using ViennaRNA (31) in 71-nt windows, and two non-overlapping low-structure windows were picked as subsections along with a window close to the 5′ end. In the case of natural RNA-triggered switches containing a ‘linker’ sequence, switches were designed using NUPACK as described above, with default stop conditions. Design code for the NUPACK web server is available in Supplementary Text S1 and S2.

### *In vitro* assay for gRNA switch processing

Oligos encoding a T7 promoter and switch or trigger sequence were ordered from IDT for PCR amplification. Amplicons were purified and concentrated using a DNA Clean & Concentrator kit (Zymo Research, D4014) for use as a DNA template for *in vitro* transcription. RNA was transcribed using the HiScribe T7 High Yield RNA Synthesis Kit (New England Biolabs, E2040S) and treated with Turbo DNase (Life Technologies, AM2238) according to the manufacturer's instructions. The RNA was resolved on an 10% polyacrylamide gel (20 × 20 cm) containing 7 M urea at 300  V for 210  min, stained with SYBR Green II (Biozym, Art.-Nr.: 850523), excised and extracted using the ZR small-RNA PAGE Recovery kit (Zymo Research, R1070) in nuclease-free water according to the manufacturer's instructions. The resulting RNAs were individually boiled in a thermocycler at 95°C for 10 min, cooled down to room temperature, and kept on ice. The processing reaction was set up by adding 357 nM of switch, 357 nM of trigger, and 510 nM of purified FnCas12a protein (Applied Biological Materials, K087) in the supplied reaction buffer. After incubation for 60 min at 37°C, the reaction was stopped by adding RNA loading buffer (0.025 bromophenol blue, 0.025% SDS, 0.025% xylene cyanol, 18 mM EDTA (pH 8.0), 93.64% formamide) on ice. Then the mixture was boiled in a thermocycler at 95°C for 10 min, resolved on an 10% polyacrylamide gel (20 × 20 cm) containing 7-M urea at 300 V for 210  min, stained with SYBR Green II (Biozym, Art.-Nr.: 850523) and visualized using a Phosphorimager (Typhoon FLA 7000, GE Healthcare). The Low Range ssRNA Ladder (New England Biolabs, N0364S) was used as the size marker.

### Preparation of cell-free TXTL reactions

*Escherichia coli* TXTL reaction mixtures were sourced commercially (Arbor Biosciences, Cat: 507024). Each 75 μl, 1.25×-concentrated, MyTXTL reaction was loaded with the necessary DNA expression templates and ultimately divided into 5 μl individual reaction droplets for incubation, expression and kinetic analysis. To prepare reactions, the 75 μl MyTXTL lysate was first thawed on ice. Reagents shared across every reaction aliquot were added first to prepare a master mix. At this stage, using highly concentrated reagents is possible without introducing pipetting inaccuracies and helps to avoid over-dilution. This TXTL master mix was lightly vortexed and centrifuged at low speed before returning to ice. In some cases, where specified, a short incubation at 29°C was used to pre-express certain components (e.g. 2-h pre-expression of dFnCas12a as described below) prior to addition of the reporter DNA template and any other experimental DNA templates. Fluorescence reporter DNA was then added to the TXTL master mix, vortexed and finally centrifuged. The TXTL master mix was then divided into smaller aliquots between 12 and 20 μl in PCR tubes on ice. Different plasmid or linear DNA templates specific to each experiment were added to these aliquots. The reaction aliquots were then lightly vortexed and centrifuged at low speed before returning to ice. Poor mixing at this stage can lead to variability within replicates. Finally, the reaction mixture from these PCR tubes was divided into 5 μl droplets in a 96-well V-bottom plate (Corning Costar 3357) and covered with a cap mat. The 96-well plate with TXTL droplets was loaded into a BioTek Synergy H1 plate reader at 29°C without shaking. Fluorescence of TXTL reaction was measured at Exc. 485 nm, Em. 528 nm every 3 min, for at least 16 h.

### *In silico* analysis of cross-talk between gRNA switches and RNA triggers

All switch and trigger combinations were co-folded pairwise using the NUPACK analysis package (29), a temperature 37°C, and a concentration 1 μM. The predicted equilibrium concentrations of each complex used to calculate the ratio of switch-trigger complexes compared to the total switch concentration.

### Testing of gRNA switches in cell-free TXTL reactions

TXTL reactions were prepared according to the general protocol outlined above in ‘Preparation of cell-free TXTL reactions’. We synthesized linear dsDNA expression templates as gBlocks from IDT, which included BsaI and BsmBI restriction sites if cloning and further *in vivo* testing was desired. These gBlocks are solubilized in nuclease-free water to a final concentration of 40 nM stock concentration for direct use in TXTL. To prevent degradation of linear DNA templates, GamS was added to the 75 μl TXTL reaction master mix at a final concentration of 2 μM (Arbor Biosciences, Cat: 501024) (32). The dFnCas12a plasmid expression template (pCB482) was then added to the master mix to a final concentration of 4 nM, and the mixture was vortexed lightly. Expression of functional Cas ribonucleoprotein complexes in TXTL can take on the order of 1–3 h (33). Accordingly, the mastermix was incubated for 2 h without shaking at 29°C to allow for expression of dFnCas12a prior to adding experimental and reporter DNA. For *araB* mRNA-responsive switch testing, 0.2 nM of the T7 RNAP plasmid was added as a shared reagent to facilitate expression of the *araB* mRNA trigger controlled by a T7 promoter. The p70a-deGFP (pCB556) reporter plasmid targeted by the different gRNAs and gRNA switches was added to the master mix to a final concentration of 0.5 nM. The master mix was then lightly vortexed and centrifuged. deGFP is a shorter half-life version of eGFP (34). The p70a promoter of deGFP being the target for binding and repression by dFnCas12a and the gRNA or gRNA switch, except for Sw-12 which targets the 5′ UTR of deGFP. The TXTL master mix was then divided into smaller 12–20 μl aliquots. 1 nM of linear DNA template encoding a gRNA switch or control and 4 nM of linear DNA template encoding RNA trigger was added to each aliquot. Synthetic and RyhB RNA triggers were expressed from linear dsDNA gBlock templates, while the araB mRNA or lacZ (control) mRNA triggers were expressed from a purified genome-derived amplicon with a T7 promoter appended upstream. The TXTL reactions were mixed and loaded into the plate reader as outlined above.

The fluorescence of TXTL reactions on the BioTek Synergy H1 was measured at Exc. 485 nm, Em. 528 nm every 3 min, for at least 16 h at 29°C without shaking. Often, only the endpoint fluorescence was reported in the figures. Fluorescence values for switches were normalized to the dynamic range of the corresponding Cas12a gRNA (same guide, but no switching domain). The normalized fluorescence was calculated by taking the difference in fluorescence between the switch and the targeting gRNA control divided by the difference in fluorescence between a targeting and non-targeting Cas12a gRNA control. This calculation is also captured by the following equation:

$$\begin{eqnarray*}&& {\rm Normalized}\ {\rm Fluorescence} \nonumber\\ && \quad = \frac{{{{\rm Fluor}_{{\rm expt}\ }}\ - \ {{\rm Fluor}_{{\rm Targeting}\ {\rm gRNA}}}}}{{{{\rm Fluor}_{{\rm Non}\hbox{-}{\rm Targeting}\ {\rm gRNA}\ }} - \ {{\rm Fluor}_{{\rm Targeting}\ {\rm gRNA}}}}}\end{eqnarray*}$$

### Synthetic trigger gRNA switch testing in *E. coli*

Plasmids expressing P70a-deGFP (pCB705) and dFnCas12 (pCB482) were co-transformed into *E. coli* MG1655 by electroporation. After electroporation, recovery and plating on LB-agar plus antibiotics, a single colony was picked for further transformation. Next, the plasmids containing the gRNA or gRNA switch plus synthetic trigger were transformed, resulting in MG1655 expressing deGFP, dFnCas12a, a Cas12a gRNA or gRNA switch and an RNA trigger. Three replicate colonies from each plasmid set were picked and inoculated into a deep-well plate with LB medium plus antibiotics and cultured overnight at 37°C. The next day, cultures were diluted 1:100 in 200 μl fresh pre-warmed LB media plus antibiotics within a black costar clear flat bottom assay plate (Corning 3603). The plate was loaded into a BioTek Synergy H1 plate reader shaking at 482 double-orbital rpm and 37°C. Fluorescence (Exc. 485 nm, Em. 528 nm) and optical density (ABS_600_) were measured once every 5 min.

### RyhB-responsive gRNA switch characterization in *E. coli*

*Escherichia coli* MG1655 cells were co-transformed by electroporation with dFnCas12a (pCB482) and deGFP (pCB705) expressing plasmids. Resulting strains were further transformed with gRNA switch plasmids by electroporation and plated on LB-agar plus antibiotics. For each resulting strain, three colonies were picked and inoculated into a deep well plate shaking overnight. The next morning, cultures were back-diluted 1:100 in LB media with antibiotics plus 100 μM of FeSO_4_. Diluted cells were cultured in deep-well plates for ∼3.5 h until an ABS_600_ of ∼0.5, reflecting mid-exponential growth phase. Cells were once again back diluted 1:10 in LB media with antibiotics plus 100 μM of FeSO_4_ plus varying amounts of the iron chelator 2,2’-bipyridyl (Sigma, D216305, CAS: 366-18-7) to trigger induction of RyhB expression (35). Diluted cells were cultured in deep-well plates for ∼4 h until an ABS_600_ of ∼0.6. Cells were then prepared for flow cytometry as outlined previously. As part of flow cytometry analysis, mean fluorescence values were extracted. Fluorescence values for gRNA switches were background subtracted from the fluorescence of an *E. coli* MG1655 control culture. Fluorescence values for gRNA switches were then normalized to fluorescence values for gRNA targeting and non-targeting controls, as was done with measurements from TXTL.

$$\begin{eqnarray*}&& {\rm Normalized}\ {\rm GFP}\ {\rm fluorescence} \nonumber\\ && \quad = \frac{{{{\rm Fluor}_{{\rm expt}\ }}\ - \ {{\rm Fluor}_{{\rm Targeting}\ {\rm gRNA}}}}}{{{{\rm Fluor}_{{\rm Non}\hbox{-}{\rm Targeting}\ {\rm gRNA}\ }} - \ {{\rm Fluor}_{{\rm Targeting}\ {\rm gRNA}}}}}\end{eqnarray*}$$

### *araB* mRNA-responsive gRNA switch characterization in *E. coli*

Testing of araB-responsive gRNA switches responding to plasmid-borne araB was performed in *E. coli* MG1655 Δ*araBAD* (SPC295) to ensure only a single source of trigger molecule was present. Sensing of genome-encoded *araB* was performed in *E. coli* MG1655. *E. coli* MG1655 (+/–) Δ*araBAD* was co-transformed by electroporation with p70a-deGFP (pCB705) and dFnCas12a alone (pCB482) or dFnCas12a with constitutively expressed *araB* or *lacZ* (SPC1191, SPC1192). After recovery and plating on LB-agar plus antibiotics, colonies were picked for further electroporation with the gRNA switches and gRNA controls. After recovery and plating, three replicate colonies were picked for each plasmid set and inoculated into a deep-well plate with minimal medium (1 × M9 salts, 2 mM MgSO_4_, 0.1 mM CaCl_2_, 10 μg/ml thiamine hydrochloride) containing 0.2% casamino acids plus antibiotics. For plasmid-expressed RNA triggers, 0.4% (w/v) glucose was added to the cultures. For genome-expressed RNA triggers, 0.4% glucose was added to the control culture, and 0.4% arabinose was added for araB induction and detection. The next morning, cultures were back diluted 1:100 in 200 μl of minimal media and 0.4% (w/v) glucose or l-arabinose (carbon source unchanged) into a black costar clear flat bottom assay plate with lid. The plate was loaded into a BioTek Synergy H1 plate reader at 37°C and shaking at 482 double-orbital rpm while reading ABS_600_ and fluorescence every 5 min. Cells were cultured until an ABS_600_ of ∼0.4 was reached. Cultures were then diluted to an ABS_600_ of ∼0.02 in fresh media and cultured in the plate reader reading ABS_600_ and Fluorescence every 5 min for 6 h. ABS_600_ was plotted on a log scale and a 3.5-h window of time where ABS_600_ increased linearly, corresponding to exponential growth phase, was isolated for further analysis. An analog measure for GFP synthesis rates on a per-cell basis was then calculated as the change in fluorescence divided by the change in ABS_600_, as described previously (36). Differentials were calculated over 20-min (i.e. 4-data-point) time windows. These differentials were calculated throughout the 3.5-h time window with the endpoint data excluded (first and last 20 min). The differential is expected to be flat during exponential growth phase, meaning that deGFP production rate is at a pseudo steady-state. The mean differential was calculated between the three replicates, using a single representative time point. The mean of differentials for the gRNA switches was then normalized against the same differential for the non-targeting and targeting gRNA controls. This gives a ‘normalized GFP expression’ which is then plotted in a similar fashion to previous TXTL and *in vivo* experiments in this work.

$$\begin{equation*}{\rm Differential}\ = \ \frac{{{{\rm Fluor}_{t2}} - {{\rm Fluor}_{t1}}}}{{{\rm ABS}{{600}_{t2}} - {\rm ABS}{{600}_{t1}}}}\end{equation*}$$

$$\begin{eqnarray*}&& {\rm Normalized}\ {\rm GFP}\ {\rm expression} \nonumber\\ && \quad = \frac{{{{\rm Diff}_{{\rm expt}}} - {{\rm Diff}_{{\rm Targeting}\ {\rm gRNA}}}}}{{{{\rm Diff}_{{\rm Non}\hbox{-}{\rm Targeting}\ {\rm gRNA}}} - {{\rm Diff}_{{\rm Targeting}\ {\rm gR1NA}}}}}\end{eqnarray*}$$

### Statistical analyses

We utilized a two-tailed t-test assuming unequal variances and a 95% confidence interval to assess statistical significance via MATLAB’s ttest2 function.

## RESULTS

### Implementing a dual-sensory domain with Cas12a gRNA switches uncouples RNA sensing and gRNA targeting

**Figure 1.**
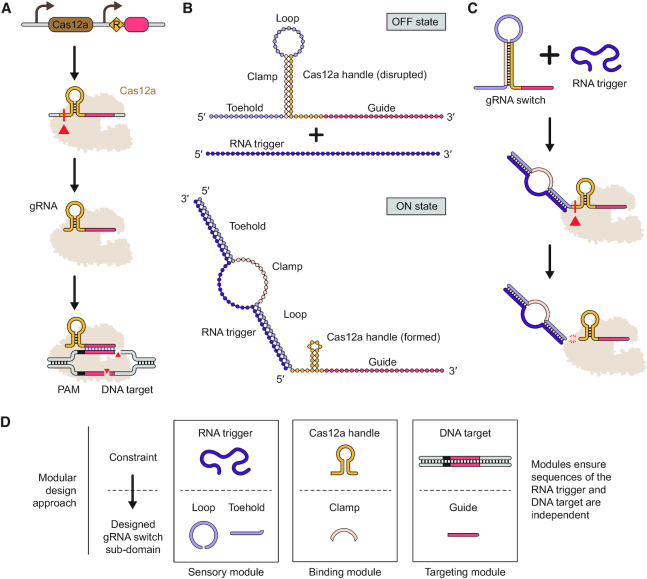
Modular design of Cas12a gRNA switches with unconstrained RNA triggers and DNA targets. (**A**) gRNA processing and DNA targeting by Cas12a. The gRNA consists of a direct repeat and spacer. The repeat forms a canonical hairpin ‘handle’ recognized by Cas12a, which cleaves upstream of the hairpin. The downstream portion of the repeat and the spacer are retained as the processed gRNA utilized by Cas12a for targeted cleavage of complementary DNA sequences flanked by a PAM. (**B**) Architecture and intended conformational states of the gRNA switch. In the absence of an RNA trigger (OFF state), the clamp base pairs with the Cas12a handle, disrupting its formation and subsequent recognition by Cas12a. In the presence of an RNA trigger (ON state), the toehold and loop base pair with the RNA trigger, leading to an energetically favorable conformation wherein the Cas12a handle re-folds and can be recognized by Cas12a. The split sensor domain does not overlap with the clamp or the guide sequences, resulting in sequence independence of the trigger, Cas12a handle and guide sequences. Both conformational states as well as the desired guide are supplied to NUPACK to design the trigger, toehold and loop. (**C**) Switch activation and processing in the presence of an RNA trigger. Hybridization between the RNA trigger and the gRNA switch drives a conformational change that allows formation of the Cas12a handle. Handle recognition leads to gRNA switch processing via Cas12a's inherent endoribonuclease activity. (**D**) The modular design approach for gRNA switches. Each module links a design constraint with a corresponding switch sub-domain.

We sought to exploit mechanisms of Cas12a crRNA biogenesis to render gRNA activity dependent on sensing of a separate ‘trigger’ RNA. A standard gRNA comprises a direct repeat upstream of a target-specific guide sequence. Once transcribed, the repeat folds into a ‘handle’ comprising a hairpin and pseudoknot recognized by Cas12a (37), resulting in Cas12a cleaving immediately upstream of the handle through the Cas12a's endoribonuclease domain (38). The upstream portion of the repeat is discarded, while the downstream portion of the repeat and the guide are retained as a gRNA (Figure 1A). Therefore, the folding requirements of the handle offered an opportunity to render Cas12a recognition of the gRNA dependent on hybridization to a separate trigger RNA. Furthermore, the endoribonuclease processing of the hairpin by Cas12a could remove upstream sequences appended to the 5′ end of the gRNA which might interfere with Cas12a function.

To take advantage of both intrinsic functions of Cas12a, we appended an artificial switching domain to the 5′ end of the processed gRNA (19-nt processed repeat and 24-nt guide) that would only allow formation of the Cas12a handle upon base pairing with a separate trigger RNA (Figure 1B). The switching domain comprises three modular sub-domains: a 15-nt toehold, a 13-nt clamp and a 15-nt loop. The clamp base pairs with the handle, disrupting hairpin formation and recognition by Cas12a. The toehold and loop base pair with the RNA trigger, driving formation of an alternative RNA structure in which the clamp can no longer sequester the handle. The resulting 86-nt gRNA switch therefore would fold into two discrete conformational states: an OFF state associated with low Cas12a activity in the absence of the RNA trigger, and an ON state associated with high Cas12a activity in the presence of the RNA trigger. In the ON state, the gRNA would further undergo processing by Cas12a, resulting in a standard gRNA free of the artificial switching domain (Figure 1C).

A key feature of the switch architecture is the lack of inherent constraints on the sequence of the RNA trigger or the guide (Figure 1D). Sequence flexibility was achieved by splitting the sensory domain responsible for RNA-trigger binding into the toehold and the loop and placing them on either side of the clamp. This configuration ensures mutually exclusive OFF and ON states without overlapping sequence constraints placed on the toehold, loop and the clamp. As a result, the RNA trigger and the guide can be selected independently and without consideration of the repeat handle sequence, in contrast to many prior gRNA switch designs (20–22). Each switch is designed by supplying the RNA folding and design algorithm NUPACK (29,30) with the two conformational states as well as the fixed repeat and the desired guide sequence (see Methods and Text S1 and S2).

### RNA triggers drive gRNA switch processing and dFnCas12a-mediated gene repression in TXTL and in *E. coli*

To test this design scheme, we first generated two switches (Sw-1, Sw-2) and cognate synthetic RNA triggers (Tr-1, Tr-2) to function with the well-studied Cas12a from *Francisella tularensis* subsp. *novicida* U112 (FnCas12a) (39). Both switches were computationally designed with guides targeting the same sequence in the p70a promoter controlling deGFP expression in a reporter plasmid and respond to distinct 43-nt synthetic RNA triggers. To characterize the DNA targeting by the switches, we relied on transcriptional repression with a nuclease-inactive version of FnCas12a (dFnCas12a) used previously for gene repression (40,41).

We initially evaluated the capacity of the RNA triggers to drive processing of the gRNA switches by FnCas12a under *in vitro* conditions (Supplementary Figure S1). *In-vitro* transcribed switches Sw-1 and Sw-2 were incubated with their cognate RNA trigger or a randomized RNA trigger in the presence of purified FnCas12a, and the RNAs were resolved on a denaturing gel. Sw-2 yielded a band at the size expected for a processed gRNA only in the presence of its cognate RNA trigger, in line with the RNA trigger driving processing by FnCas12a. Sw-1 exhibited a faint band under all conditions involving FnCas12a, suggesting that a subpopulation of the Sw-1 RNA might adopt the ON state even in the absence of its RNA trigger.

**Figure 2.**
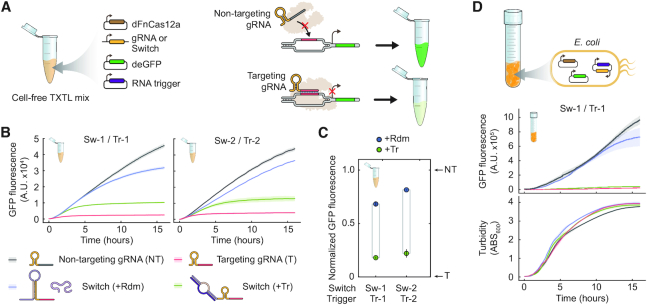
Cas12a gRNA switches enable RNA-triggered DNA targeting in TXTL and in *E. coli*. (**A**) Assessing gRNA switch activity in TXTL. DNA encoding dFnCas12a, a gRNA or gRNA switch, an RNA trigger and the deGFP reporter were combined in the reaction, and fluorescence is tracked over time. DNA targeting by dFnCas12a would result in reduced fluorescence. (**B**) Fluorescence time courses from TXTL. The central line represents the average while the surrounding band represents the standard error from three independently mixed reactions. (**C**) End-point measurements from the TXTL assays in B as assessed at 16 h. Fluorescence values were normalized so the targeting and non-targeting gRNAs yield 0% and 100% GFP fluorescence, respectively. (**D**) Assessing gRNA switches in *E. coli*. *E. coli* cells harboring plasmids encoding the same components from the TXTL assay were monitored for fluorescence and turbidity over time. All components were constitutively expressed. See B for the legend. The central line represents average while the surrounding band represents the standard error from three independent experiments starting from separate transformants.

We next characterized DNA targeting by the switches using a cell-free transcription-translation system (TXTL) derived from *E. coli* (42), which we previously employed to rapidly characterize CRISPR technologies (33,43). When using TXTL, DNA encoding all of the components is added to the reaction, leading to their transcription and, for protein products, translation. Fluorescence from the expressed deGFP reporter is measured over time, serving as a quantitative and dynamic readout of FnCas12a activity (Figure 2A). As part of these assays, a targeting (T) and non-targeting (NT) gRNA established the limits of gRNA-directed activity (0% and 100% deGFP fluorescence), while the randomized RNA trigger (+Rdm) established the leakiness of the switch (Figure 2B). At the end of the reaction time course, Sw-1 and Sw-2 respectively yielded high fluorescence (68% and 81% normalized deGFP fluorescence) with the randomized RNA trigger and low fluorescence (18% and 22% normalized deGFP fluorescence) with the cognate RNA trigger, respectively (Figure 2B-C). The reduced fluorescence in the presence of the cognate RNA trigger is in line with activation of the gRNA switch and reporter silencing by dFnCas12a, where Sw-1 and Sw-2 respectively achieved 50% and 59% of dFnCas12a's dynamic range. The higher basal activity exhibited by Sw-1 is in line with the results from the *in vitro* processing assay (Supplementary Figure S1). The observed silencing activity was dependent on dFnCas12a, as swapping dFnCas12a with dSpyCas9 resulted in high fluorescence for all gRNAs (Supplementary Figure S2). In addition, the gRNA switches also yielded RNA triggered silencing with FnCas12a, demonstrating that the gRNA switches can also control DNA cleavage (Supplementary Figure S3). The Cas12a gRNA switches therefore function as designed, at least in the context of cell-free systems.

Finally, we evaluated the gRNA switch designs *in vivo* by constitutively expressing the same components and the deGFP reporter from plasmids in *E. coli* MG1655. Sw-2 and Tr-2 could not be cloned together into one expression plasmid, although Sw-1 and Tr-1 were successfully cloned and tested (Figure 2D). Beyond its performance in TXTL, the Sw-1 and Tr-1 pair exhibited negligible basal activity and triggered activity approaching that of the targeting gRNA in *E. coli*, with 6% and 79% GFP production in the presence or absence of the RNA trigger, respectively, and an overall fold-change of ∼12 during late log-phase growth. The fold-change in switch activation observed *in vivo* was more than three times higher than in TXTL. This difference can be explained by the build-up of deGFP in TXTL prior to dCas12a:gRNA complex formation and DNA targeting. Despite this distinct feature of TXTL, previous work demonstrated a correlation between the magnitude of transcriptional repression in TXTL and *E. coli* using Cas9, and a similar correlation is expected for Cas12a (33). Therefore, Cas12a gRNA switches can function *in vivo* and may have higher fold activation than observed in TXTL.

### gRNA switches respond to different RNA triggers and operate orthogonally

**Figure 3.**
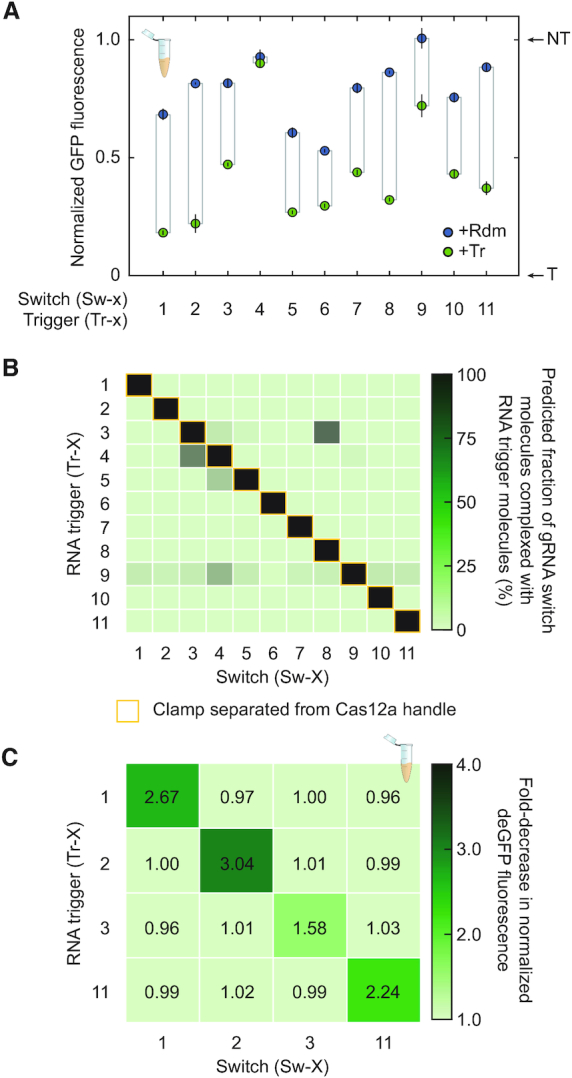
An expanded set of gRNA switches respond to their synthetic RNA triggers without crosstalk. (**A**) Assessing DNA targeting activity of an expanded set of gRNA switches that respond to synthetic RNA triggers in TXTL. See Figure 2C for details. (**B**) NUPACK predictions for cross-talk between gRNA switches and their artificial RNA triggers. Predictions are based on co-folding each gRNA switch and RNA trigger each at a simulated concentration of 1 μM. (**C**) Evaluating cross-talk between the subset of gRNA switches and RNA triggers in TXTL. Fold-increase in normalized deGFP fluorescence is reported as the average from three independently mixed reactions. See Figure 3C for details on the normalization.

We next expanded the set of tested gRNA switches and evaluated the extent of cross-talk between the artificial triggers. We used NUPACK to design nine additional switches triggered by 43-nt long synthetic RNA triggers and targeting the same location in the promoter controlling deGFP. All switches were designed with a 15-nt long toehold, 13-nt clamp, and a 15-nt loop structure, and the GC content of the triggers varied from 30% to 53%. In TXTL, all but one of the switches (Sw-4) yielded a significant decrease in deGFP fluorescence in the presence of the RNA trigger versus the randomized RNA trigger (*P* = 0.5 for Sw-4 and *P* = 5.0 × 10^−5^ – 0.012 for the other switches, *n* = 3). Functional switches maintained 16–59% of the dynamic range set by the targeting and non-targeting gRNAs (Figure 3A). DNA targeting activities also varied widely under both triggered and untriggered conditions, with triggered normalized GFP fluorescence varying between 0% and 47% and untriggered normalized GFP fluorescence varying between 27% and 82%. Therefore, changing the trigger sequence and the resulting sensory domain affects switch performance.

We next asked to what extent cross-talk can occur between the gRNA switches by comparing switch activity with the cognate trigger against every other trigger. We first employed NUPACK to predict the extent to which an RNA trigger would complex with the gRNA switch and release the clamp from the Cas12a handle (Figure 3B). All gRNA switches fully complexed with their cognate RNA trigger, as expected based on switch design. In contrast, only 10.6% (12/110) of the switch:non-cognate trigger pairs were predicted to form complexes comprising over 5% of the population of each gRNA switch at equilibrium. Furthermore, and most importantly, the clamp was always predicted to be paired with the Cas12a handle for all minimal free energy secondary structures across the non-cognate switch:trigger pairs.

To evaluate the extent of cross-talk experimentally, we applied the TXTL assay to evaluate four of the switches (Sw-1, Sw-2, Sw-3, Sw-11) that exhibited medium to high dynamic ranges and were not predicted to be activated by each others’ RNA triggers in TXTL (Figure 3C). In line with the NUPACK predictions, none of the non-cognate switch:trigger pairs registered a statistically significant decrease in normalized deGFP fluorescence compared to using the randomized RNA trigger (*P* = 0.16–0.92, *n* = 3). We also evaluated the interaction between Sw-3 and Tr-8, the strongest predicted for non-cognate pairs. Negligible switching was observed in TXTL by Sw-3 when paired with Tr-8 versus the random trigger (*P* = 0.21), in line with the lack of a properly folded Cas12a handle in the predicted switch:trigger complex (Supplementary Figure S4). Therefore, the gRNA switches can respond orthogonally to different RNA triggers.

As all gRNA switches targeted the same location in the promoter of the deGFP reporter construct, we designed and tested an additional switch (Sw-12) that responds to a distinct RNA trigger and targets the 5′ UTR immediately upstream of the *deGFP* start codon (Supplementary Figure S5). The associated gRNA was less efficient at silencing deGFP expression compared to the gRNA targeting the promoter, although the associated gRNA switch exhibiting robust activity, with normalized deGFP fluorescence of 32% and 74% in the presence and absence of the RNA trigger, respectively. Therefore, the design scheme for gRNA switches can accommodate different guide sequences and DNA targets.

### The endogenous iron-stress response of *E. coli* can be linked to dCas12a activity via an optimized gRNA switch

**Figure 4.**
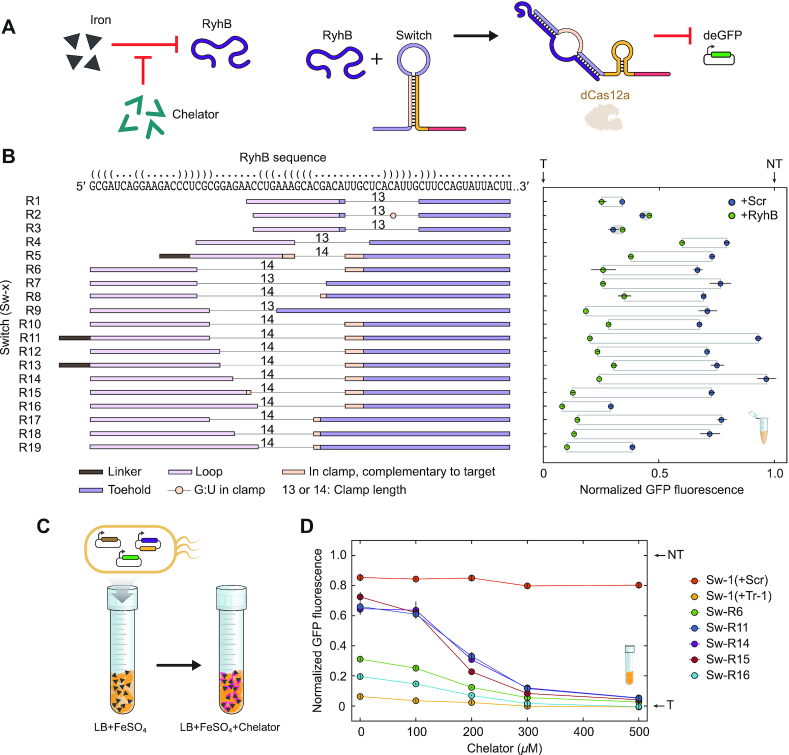
Switch optimization allows robust sensing of the Hfq-binding small RNA RyhB in TXTL and in *E. coli*. (**A**) Activation of gRNA switches via induction of RyhB through iron starvation. RyhB is naturally induced in *E. coli* following depletion of iron in the culture, such as through the addition of the iron chelator 2,2′ bipyridyl to the growth medium. (**B**) Modifying gRNA switches to enhance sensing of RyhB in TXTL. Left: RyhB sequence (top) and RyhB switch (Sw-R1 to Sw-R19) sensor domain structures, displayed 3′ to 5′. The predicted secondary structure of RyhB is represented as dot-bracket annotation and matches that shown in Supplementary Figure S7. The toehold and loop, as well as complementary regions of the clamp, are aligned with their binding locations along the RyhB sequence. The linker does not bind RyhB. All gRNA switches targeted the same location in the promoter controlling deGFP expression. Right: end-point measurements of normalized deGFP fluorescence in the presence of RyhB or the randomized RNA trigger (+Rdm). See Figure 2C for details. (**C**) Assessing gRNA switch activity following induction of RyhB expression in *E. coli*. Different concentrations of 2,2′-bipyridyl were added to the growth medium to induce RyhB expression. (**D**) Impact of gRNA switches on deGFP expression following induction of RyhB. deGFP expression was measured by flow cytometry analysis. deGFP fluorescence was normalized to that of the targeting gRNA (0%) and the non-targeting gRNA (100%) subjected to the same growth conditions. Values represent the average and standard error of three independent experiments initiated from separate colonies.

Having confirmed the functionality of switches triggered by artificial RNA triggers, we set out to evaluate the extent to which our design platform can be implemented to sense naturally-occuring RNAs. We initially focused on Hfq-binding small RNAs (sRNAs), which base-pair with target mRNAs to exert post-transcriptional and translational regulation. Because these sRNAs evolved to form base-pairing interactions with other RNAs, their structure might be amenable to base pairing with a gRNA switch (44). As a proof-of-principle demonstration, we selected RyhB, an sRNA native to *E. coli* and other enteric bacteria involved in the iron-starvation stress response (35,45). A RyhB-responsive switch targeting the deGFP reporter is thus expected to silence deGFP expression under low-iron conditions when RyhB is expressed (Figure 4A).

We initially designed a RyhB-responsive switch, Sw-R1, with the same intended switching domain composition and guide as the prior switches. This composition included a 15-nt toehold, a 13-nt clamp, a 15-nt loop, and a guide targeting the promoter driving deGFP expression. The toehold and loop were designed to base pair in part with regions of RyhB predicted to be single-stranded (Supplementary Figure S6). We first employed the TXTL assay to test the capacity of Sw-R1 to silence deGFP expression in the presence of a constitutively-expressed RyhB trigger. We observed high basal activity with the randomized RNA trigger and only a modest reduction in deGFP fluorescence with RyhB (Figure 4B). Therefore, there was ample room for improvement from our initial switch design.

We hypothesized that the binding location within RyhB as well as the modular sensing sub-domains could be varied to enhance switch performance beyond that achieved with Sw-R1. We designed a collection of 18 new RyhB-responsive gRNA switches (Sw-R2–Sw-R19), where we gradually increased the length of the toehold, clamp, and loop as well as shifted the binding region in RyhB. One design (Sw-R2) included a G–U base pair in the clamp designed to weaken its strength. In some cases, there was some complementarity between the clamp and the target sequence. In three designs (Sw-R5, Sw-R11 and Sw-R13), we introduced a new short ‘linker’ sub-domain, a 5-nt sequence between the loop and the Cas12a handle, which does not hybridize with the trigger. The linker is designed by NUPACK to remain single-stranded in both ON and OFF states and grants the algorithm some flexibility when identifying the final switch sequence, resulting in a reduction in predicted secondary structure within the switch loop sub-domain (Supplementary Figure S7).

We then characterized the new RyhB-responsive switches in TXTL in the presence of RyhB or the randomized RNA trigger (Figure 4B). Switches with toeholds and loops shorter than 17 nts or with a weakened clamp through introduction of a G–U base pair yielded negligible to minor sensing of RyhB (0–19% of the dynamic range for the targeting and non-targeting gRNAs). However, extending the toehold and clamp rescued the activity of the gRNA switches, with 7 out of 11 switches with loop lengths of at least 19 nts retaining at least 50% of the dynamic range. The addition of a linker improved the response compared to an otherwise identical sequence in one case (R11 displayed 73% of wild-type gRNA dynamic range with the linker versus 39% for R10 without the linker) but not another case (44% of dynamic range with the linker for R13, and 47% without the linker for R12). The linker can therefore improve switch performance in some contexts. The best performing switches, R11 and R14, retained 72% of the wild-type dynamic range and had <7% leakiness when a random-sequence RNA trigger was expressed in TXTL.

We selected five RyhB-sensing switches to evaluate their functionality in *E. coli* MG1655 under low-iron conditions (Figure 4C, Supplementary Figure S3). We expected unhindered deGFP expression and high fluorescence during stress-free growth when RyhB is weakly expressed, and low fluorescence during iron starvation when RyhB is strongly expressed (35). To identify contributions from iron starvation to deGFP expression independent of dCas12a activation, we used Switch Sw-1 as a control with its cognate trigger Tr-1 or with the randomized RNA trigger. The iron chelator 2,2′-bipyridyl was added at different concentrations to the growth medium as before (35), and deGFP expression was monitored by flow cytometry analysis. We found that the RyhB-responsive gRNA switches Sw-R11, Sw-R14 and Sw-R15 exhibited striking dose-dependent repression of deGFP expression. Untriggered normalized fluorescence of R11, R14 and R15 was 66%, 64% and 72% in the absence of the chelator, and 5%, 5% and 4% in the presence of 500 μM 2,2′-bipyridyl, demonstrating the ability of the optimized switches to detect RyhB RNA *in vivo* (Figure 4D).

### gRNA switches exhibit a less efficient, but measurable, response to the mRNA encoding AraB

**Figure 5.**
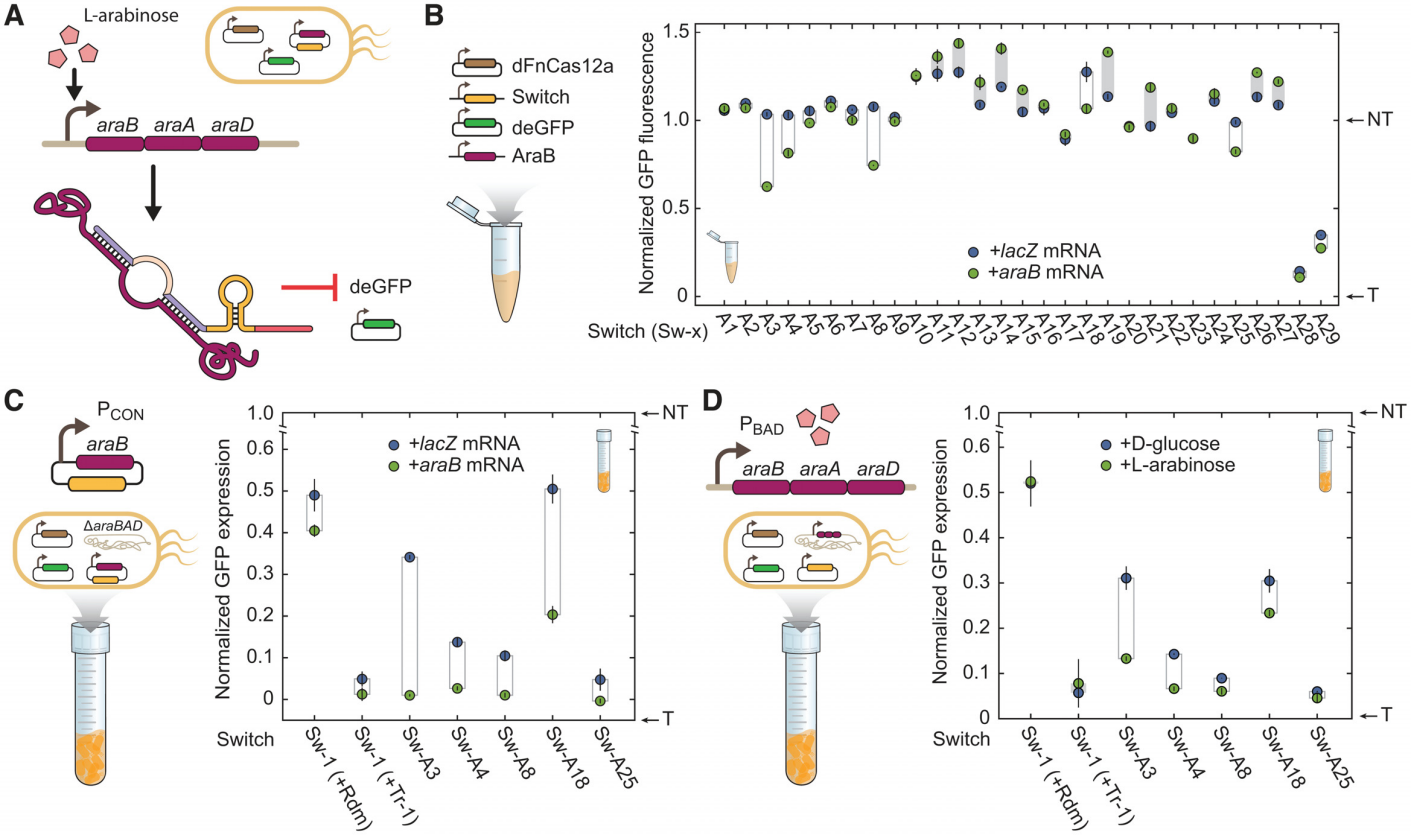
gRNA switches can be triggered by the *araB* mRNA in TXTL and in *E. coli*. (**A**) Sensing the mRNA encoded by *araBAD* to drive deGFP silencing with gRNA switches. (**B**) Assessing gRNA switches designed to sense the *araB* mRNA in TXTL. See Figure 2C for details. Locations of the trigger RNAs within the *araB* mRNA are shown in Supplementary Figure S8. Some of the gRNA switches exhibited increased fluorescence with the *araB* mRNA trigger, possibly due to differences in the size and sequence of the expressed transcripts (70). (**C**) Assessing activation of gRNA switches in *E. coli* when constitutively expressing the *araB* mRNA or a *lacZ* mRNA control. The endogenous *araBAD* operon was deleted to prevent switch activation from this locus. Growth and fluorescence measurements were made on a microtiter plate reader. GFP expression rates were calculated as differentials of fluorescence over ABS_600_ and normalized to the non-targeting (0% GFP expression rate) and targeting gRNA (100% GFP expression rate). Sw-1 with its cognate trigger (Tr-1) or a randomized RNA trigger (Rdm) served as controls. Values represent the average and standard deviation of three independent experiments initiated from separate colonies. (**D**) Assessing activation of gRNA switches when inducing expression of the endogenous *araBAD* operon. *E. coli* cells were induced with the addition of L-arabinose or repressed with the addition of D-glucose to the medium. See C for details.

Having optimized gRNA switches to sense a short Hfq-binding sRNA, we asked if our designs could be extended to sense long mRNAs. We selected the mRNA encoding the *araBAD* operon involved in L-arabinose catabolism in *E. coli* as a proof-of-principle demonstration. Transcription of the *araBAD* mRNA is strongly upregulated uniformly across the cell population in the presence of high concentrations of L-arabinose (Figure 5A) (46). We designed a series of switches targeting the promoter controlling deGFP expression and triggered by different sequences within the mRNA encoding *araB*. The designed switches harbored varying sub-domain lengths and compositions, including toeholds of either 24 nts or 30 nts, clamps between 14 nts and 16 nts, and loops between 23 nts and 27 nts. Leveraging the lack of constraints on the selected trigger, we selected trigger sequences from three different regions of the mRNA at the 5′ end or predicted to exhibit weak local secondary structures (Supplementary Figure S8, Supplementary Table S4). The three trigger sequences were spaced out across the length of the *araB* ORF, with Sw-A1 through Sw-A9 near the 5′ end of the ORF, Sw-A10 through Sw-A19 about 25% into the ORF, and Sw-A20 through Sw-A29 near the 3′ end of the ORF (Supplementary Figure S8, Supplementary Table S4).

We initially screened our *araB*-responsive switch candidates in TXTL by constitutively expressing the full *araB* coding sequence in the presence of our switches, dFnCas12a, and our reporter deGFP (Figure 5B). Five of the switches screened (Sw-A3, Sw-A4, Sw-A8, Sw-A18, Sw-A25) displayed statistically significant activation in the presence of *araB* mRNA compared to a *lacZ* mRNA control (*P* = 5.3 × 10^−4^ – 0.05, *n* = 3). Notably, Switch Sw-A3 retained 41% of the dynamic range of the targeting and non-targeting gRNAs. Other switches, Sw-A4, Sw-A8, Sw-A18 and Sw-A25 retained between 17% and 33% of the gRNA control dynamic range. Unexpectedly, several switches displayed increased fluorescence in the presence of their cognate trigger. Out of the three trigger sequence regions within *araB*, the region near the 5′ end of the ORF yielded the highest frequency of active switches (Figure 5B, Supplementary Figure S4). We did not observe any clear trends in activity when varying toehold, clamp and loop lengths. We thus were able to generate *araB* mRNA-responsive gRNA switches, although the performance was generally lower than those for the RyhB-responsive switches.

We next assessed the five functional *araB* mRNA-responsive switches in *E. coli* MG1655 by measuring deGFP fluorescence. In this case, cells were cultured in micro-well plates, and fluorescence was measured over time on a plate reader to streamline the experiments. We initially tested the switches in an *araBAD*-deletion mutant constitutively expressing the *araB* mRNA or a *lacZ* mRNA control from a low-copy plasmid (Figure 5C). Sw-1 was again used as a control to detect effects of the *lacZ* or *araB* mRNA on reporter expression independent of switch interactions. Sw-1 paired with the random trigger exhibited non-significant activation in presence of an *araB* mRNA as opposed to a *lacZ* mRNA (*P* = 0.098). We found that switches Sw-A3 and Sw-A18 displayed the highest switching activity, with 33% and 30% of the dynamic range for the targeting and non-targeting gRNA controls. Switches Sw-A4, Sw-A8 and Sw-A25 displayed modest but significant activation (*P* = 0.0047–0.013, *n* = 3). We also noted that Sw-1 exhibited reduced activation than in previous trials (dynamic range of 39% versus 73% and 79% in Figures 2D, 4D), indicating that the strain itself or use of minimal medium affected switch performance. Nevertheless, the *araB* mRNA-dependent activation of Sw-A3 and Sw-A18 demonstrate that Cas12a gRNA switches can be used to sense mRNA *in vivo* in *E. coli*.

We finally assessed the functionality of the *araB* mRNA-responsive gRNA switches in *E. coli* when inducing expression of *araBAD* in its native context. Here, we expressed dFnCas12a, the gRNA switches and the deGFP reporter from plasmids, and we induced or repressed expression of the *araBAD* operon through the addition of l-arabinose or D-glucose to the growth medium (Figure 5D). Strains harboring Sw-A3 and Sw-A4 exhibited significantly higher fluorescence when grown with d-glucose than with l-arabinose (*P* = 0.019 and 0.0024 respectively, *n* = 3). D-glucose and l-arabinose had a negligible effect on the fluorescence levels of Sw-1 controls (*P* = 0.56, 0.71, *n* = 3), indicating that the switching activity observed by our *araB* mRNA-responsive switches is not due to an indirect metabolic effect. The activation was lower than when sensing the plasmid-expressed *araB* mRNA, although the best-performing switch (Sw-A3) still retained 17% of the dynamic range for the targeting and non-targeting gRNA controls. Switches can therefore be used as sensors of the cellular transcriptional state mediated by a long mRNA trigger, and they can convert this signal into activation of a CRISPR effector.

## DISCUSSION

We report a distinct architecture for engineering Cas12a gRNAs activated by an RNA-of-interest (Supplementary Figure S9). Switches fold into an inactive state in isolation but are activated in the presence of an RNA trigger. Sensing of the RNA trigger results in unfolding of the switch, processing by Cas12a, and gRNA-directed DNA targeting activity. Switch processing results in a standard gRNA, offering an advantage over Cas9 switches that maintain the appended artificial RNA sequence that could impede proper gRNA function. Another key feature of our switch design is that the RNA-sensing sub-domains do not overlap with the clamp domain, freeing our design from sequence constraints. In other prior switch designs (20–22), the clamp structure is unfolded through strand invasion, which entails homology between the trigger and the blocked handle or the guide sequence. In turn, the forced homology either limits the range of guide targets or of RNAs that can be sensed by the switch, and it mandates the use of workarounds such as the involvement of a third strand (22). On the other hand, by dividing the RNA-sensing sub-domains into a toehold and loop which do not overlap with the clamp, we were able to create switches which are free of inherent sequence constraints. This simplified the sensing of natural RNAs and, through the variation of sub-domain length, demonstrated the first example of modulating a CRISPR gRNA switch response to a given RNA trigger. Recent work has also highlighted ways to separate the guide and trigger RNA sequences for Cas9 sgRNA switches, including a design framework paralleling the one employed here (47) or involving three separate RNAs (48).

Beyond loosening constraints, separating the toehold and loop sub-domains could expand possible designs and mechanisms for CRISPR RNA switches. For instance, this separation could facilitate the seeding of RNA–RNA interactions, as they could be initiated through either the toehold, the loop, or both. A divided sensor domain also opens the possibility of sensing non-contiguous stretches of an RNA trigger, which might be useful to avoid sequences with strong secondary structure within an mRNA. Although the advantages of this division remain to be fully explored, a previously tested ‘splinted switch’ design containing a non-contiguous sensor domain yielded a higher dynamic range than the single sensing domain switch tested in the same study (20), suggesting that splitting the sensing domain might be an advantageous strategy. We also used a short linker sequence in some designs, which in one case improved switch performance. Such linkers can be inserted before the loop or clamp sub-domains of the switch. These linker sequences can be computationally optimized to increase accessibility of the loop region or to increase the predicted stability of the clamp. Increased loop accessibility might facilitate more switch-trigger interactions, while increased clamp stability might reduce leaky activity in the OFF state. Therefore, linkers might offer a means to further enhance switch activity.

We leveraged TXTL to interrogate gRNA switch designs. Unlike experiments in *E. coli*, TXTL experiments can be completed in a single day, because DNA transformation is not required and linear DNA can be introduced. The TXTL assays are highly scalable, as each reaction is conducted in 5 μl, enabling high-throughput experimentation. Unconstrained switches naturally have a larger design space than sequence-constrained switches, increasing the potential for improvements by switch performance, but thereby requiring efficient screening technologies. We characterized 60 gRNA switch designs in TXTL with different RNA triggers and conditions totaling 152 unique reactions, each in triplicate. The clear speed and scalability of TXTL over traditional *in vitro* assays and cell-based assays explains the growing use of TXTL for prototyping different CRISPR technologies (33,41,43,49) as well as riboregulators, sensory components and genetic circuits (26,50–54).

Some switches demonstrated leaky activity in the absence of their cognate trigger. One possible explanation is the presence of unpredicted RNA structures that allow Cas12a recognition without trigger binding. For example, kinetically trapped RNA structures might transiently form accessible Cas12a handles, allowing gRNA processing before formation of the clamp. Designed gRNA switches could also form pseudoknots or other more complicated structures that could compete with the intended structure, allowing Cas12a binding in the absence of the trigger. Potential competing structures can be visualized using saliency maps generated from machine learning methods (55). These maps pointed to competing structures being more prevalent in leaky toehold switches, and similar mechanisms could be at play in gRNA switches.

Although our design permits sensing of natural RNAs, we were able to more readily detect RyhB than the *araB* mRNA. While the natural base-pairing mechanism of RyhB might facilitate its interaction with gRNA switches, the length and secondary structure of long mRNAs might also hamper interaction with switches and pose a particular challenge. Other groups have reported significant decreases in detecting longer mRNAs than their small RNA counterparts, with one noting steric hindrance due to mRNA folding as a limiting factor (22,26). The lack of trigger sequence constraints on our switches could be exploited by designing a library of switches binding to a variety of regions along the mRNA. This approach would identify the best target sites along the mRNA, and might reveal potential ‘hot spots’ for RNA sensing (56). Analysis of predicted structures and free-energy parameters would likely be a starting point for improving switch design. The integration of switch libraries with machine-learning workflows might allow for the efficient extraction of switch design principles, enhancing predictive design for a future generation of switches (56,57). These same workflows could also be effective when designing mRNA-responsive switches given the complexities of predicting how these long and actively translated RNAs fold. These mRNA-responsive switches would require testing much larger sets of switches, where TXTL could be part of a rapid testing pipeline.

The ability to design and implement Cas12a gRNA switches without inherent constraints on the selected RNA trigger or DNA target unlocks numerous possibilities for advancement of CRISPR technologies. For instance, switches could restrict nuclease function to specific cell types by responding to a tissue-specific transcript, such as a germline-expressed transcript as part of CRISPR–Cas gene drives. Switches could also regulate the expression of genes based on dynamic changes in the cell, such as when responding to stress signals as part of metabolic engineering. Conditional targeting could also be implemented as part of CRISPR-based antimicrobials to target essential genes only when a sensed antibiotic-resistance marker is present. As a final example, the expanding use of Cas12a for *in vitro* RNA detection (58) offers a unique opportunity to implement gRNA switches that introduce an extra layer of detection, or to sense both an RNA and separate DNA target for multi-input diagnostics.

Beyond the implementation of Cas12a gRNA switches, our switch design scheme could be readily adapted to other CRISPR nucleases. Different nucleases beyond Cas12a are known to process the transcribed CRISPR array into individual gRNAs through recognition of specific sequences and structures within the repeat. In particular, Cas13 recognizes a hairpin in the transcribed repeat that drives processing through an endoribonuclease domain present in the protein (59). By incorporating a sensory domain to render hairpin folding dependent on an RNA trigger, Cas13 gRNA switches could introduce context-dependent control as part of targeted gene silencing or RNA editing in eukaryotic cells (60,61) or as an additional sensing mechanism for Cas13-based diagnostics (62). Separately, Type I and III CRISPR–Cas systems rely on the Cas6 endoribonuclease to cleave a formed hairpin in the repeat as part of crRNA biogenesis, and these systems have shown promise as gene editors, antimicrobials and gene regulators (63–69). Therefore, our design framework could be used to render a wider range of CRISPR nucleases dependent on an RNA-of-interest, with the potential to expand this functionality to a much wider swath of CRISPR technologies.

## DATA AVAILABILITY

All raw data can be made available upon request.

## Supplementary Material

gkab100_Supplemental_Files
