# Supplementary material for: Sequence-independent RNA sensing and DNA targeting by a split domain CRISPR–Cas12a gRNA switch
Source: Nucleic Acids Res. 2021 Feb 22;49(5):2985–99. doi: 10.1093/nar/gkab100 (PMC7968991; doi:10.1093/nar/gkab100)
Supplement: gkab100_Supplemental_Files [file gkab100_supplemental_files.zip › Supplementary Information_resubmission.pdf]

SUPPLEMENTARY INFORMATION

Sequence-independent RNA sensing and DNA targeting by a split domain CRISPR-Cas12a gRNA switch

Scott P. Collins, William Rostain, Chunyu Liao, Chase L. Beisel

Correspondence to chase.beisel@helmholtz-hiri.de (C.L.B.)

TABLE OF CONTENTS

Supplementary Tables …………...…………...…………...…… 2

Supplementary Figures …………...…………...…………...…… 3

Supplementary Text …………...…………...…………...…… 13

Supplementary References …………...…………...…………...…… 15

SUPPLEMENTARY TABLES

Each table is a tab in the file “Supplementary Tables.xlsx”.

Table S1. Oligonucleotides and strains used in this work.

Table S2. gBlocks and plasmids used in this work.

Table S3. gRNA switch sequences and NUPACK design constraints.

Table S4. Features of araB mRNA-responsive gRNA switches and location of RNA triggers in the *araB* ORF.

SUPPLEMENTARY FIGURES

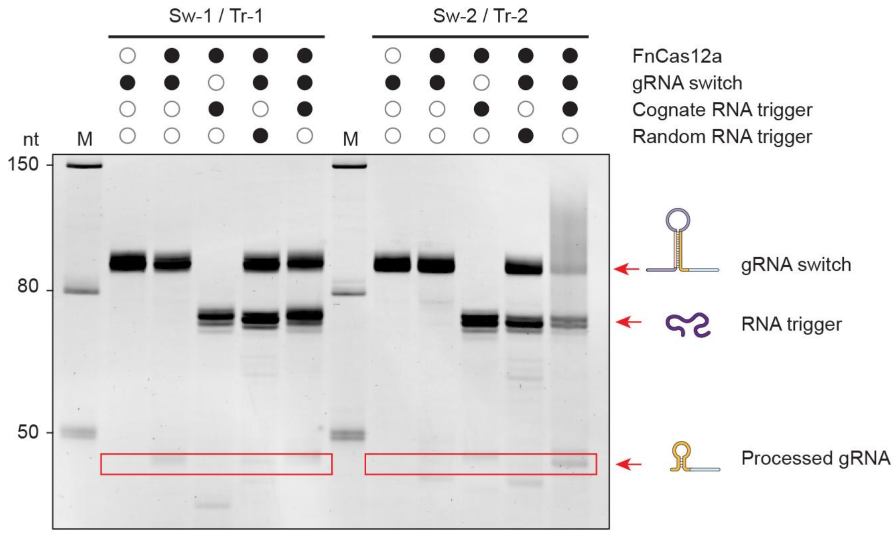

Figure S1. RNA trigger-dependent processing of gRNA switches by FnCas12a *in vitro*. The indicated combinations of FnCas12a, gRNA switch, and cognate or random RNA trigger were incubated for 60 minutes at 37°C prior to being resolved by denaturing PAGE and stained for total RNA. RNA trigger-dependent processing was more apparent for Sw-2. Expected lengths: Unprocessed switch: 86 nts, processed gRNA: 43 nts, RNA trigger (including terminator): 72 nts.

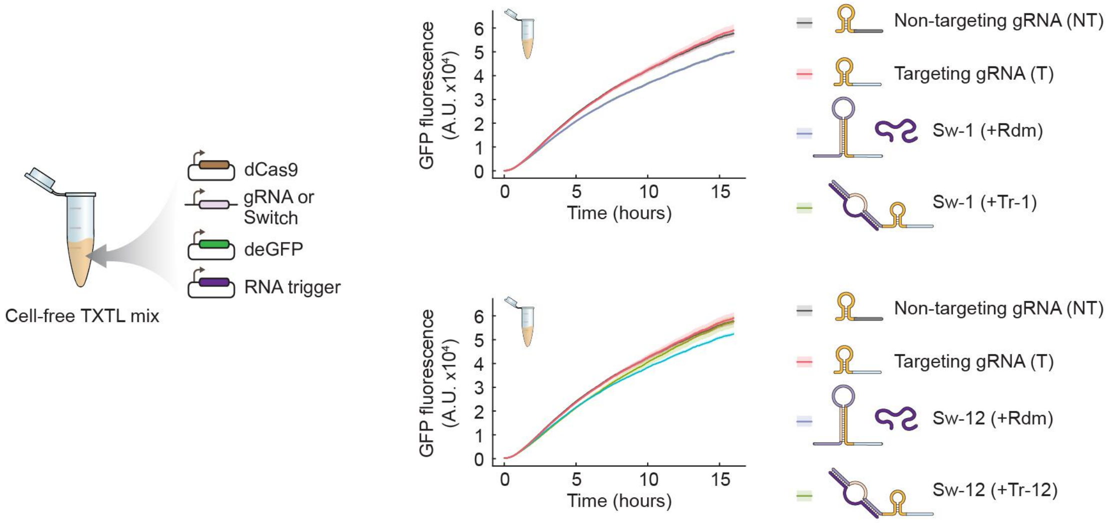

Figure S2. Fluorescence time courses from TXTL when combining the Cas12a gRNA switches with the catalytically-dead SpyCas9 (dCas9). dCas9 was used in place of dCas12a to maintain a similar burden of expression on the TXTL expression system. See Figure 2C for more information. Fluorescence was similarly high for all tested switches, confirming that Cas12a is required for switch function.

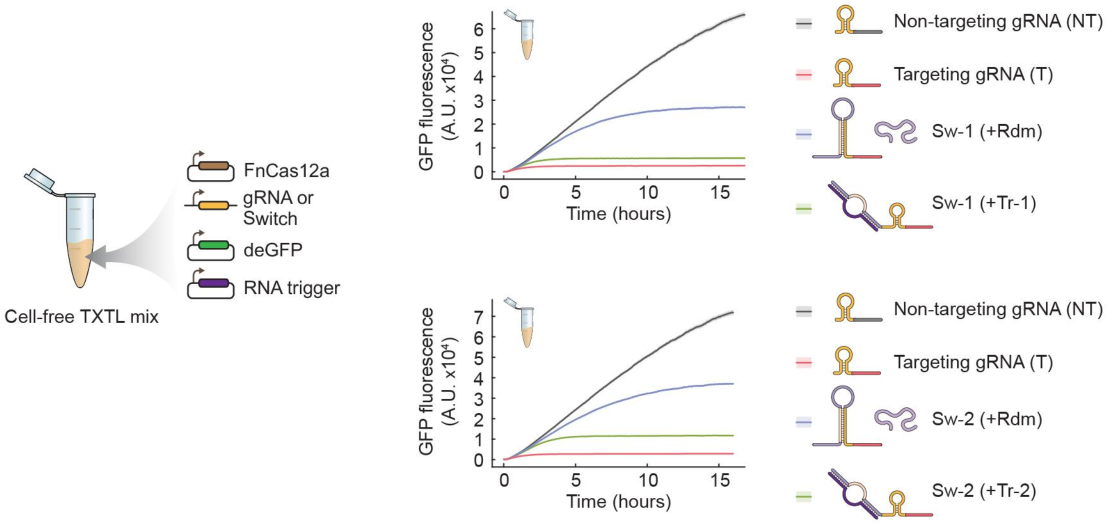

Figure S3. Fluorescence time courses from TXTL when combining the Cas12a gRNA switches with the catalytically-active FnCas12a. In this setup, Cas12a cleaved the target, resulting in loss of GFP expression. See Figure 2C for more information.

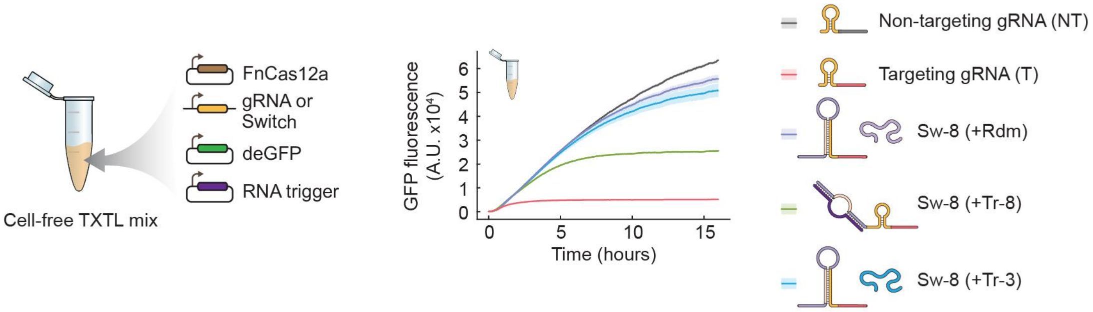

Figure S4. Fluorescence time courses from TXTL when testing cross-talk interactions between Sw-3 and Tr-8. See Figure 2C for details. The extent of GFP silencing was not statistically significant for Tr-8 compared to the random trigger (Rdm) ($p = 0.21$), in line with the lack of cross-talk predicted using NUPACK (see Figure 3B).

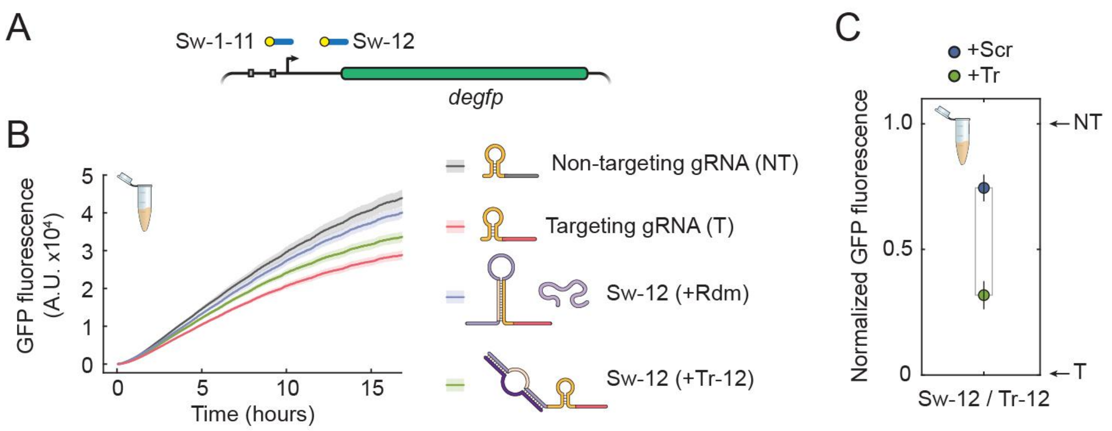

Figure S5. Functionality of Cas12a gRNA switches using a different target. (**A**) Target locations within the deGFP reporter construct. gRNA switches Sw-1 through Sw-11 targeted the same location within the constitutive sigma-70 promoter driving deGFP expression. Sw-12 targeted the template strand immediately upstream of the coding region of *degfp*, which is expected to lead to more efficient silencing than when targeting the non-template strand (1). (**B**) Assaying DNA targeting through Sw-12 in TXTL. See Figure 2B for more information. (**C**) Normalized end-point measurements from the TXTL assay in B. See Figure 2C for more information.

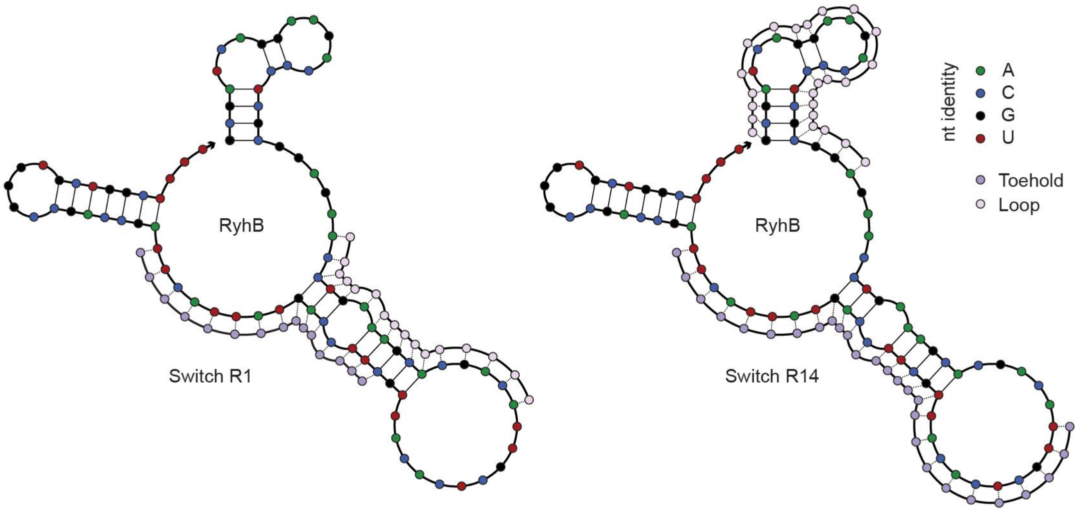

Figure S6. Predicted secondary structure of RyhB with binding locations for two RyhB-responsive gRNA switches. Secondary structures were predicted using NUPACK with the default parameters. The base-pairing locations for the toehold and loop section of the sensory domain are shown for switches Sw-R1 and Sw-R14. See Figure 4B for more details.

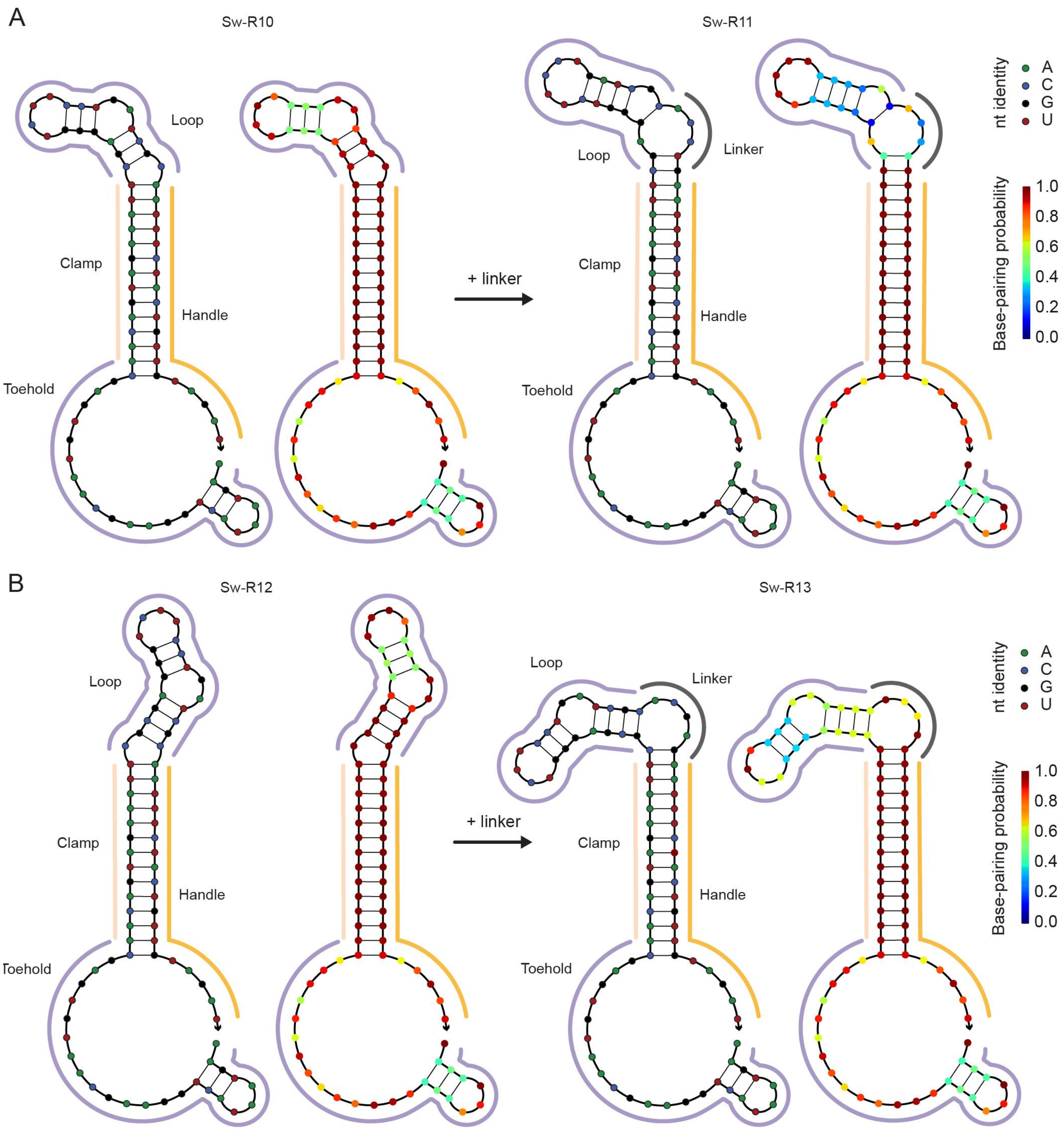

Figure S7. Impact of the added linker on the predicted folding of gRNA switches. (**A**) Addition of a linker to Sw-R10 to form Sw-R11. (**B**) Addition of a linker to Sw-R12 to form Sw-R13. The minimal free energy structure is shown for each gRNA switch, with the nucleotide identity on the left and the base-pairing probabilities on the right. In both cases, the linker was added to reduce

the extent of base pairing within the loop and extend the stem formed between the handle and the clamp. The corresponding data from the TXTL assay is shown in Figure 4B.

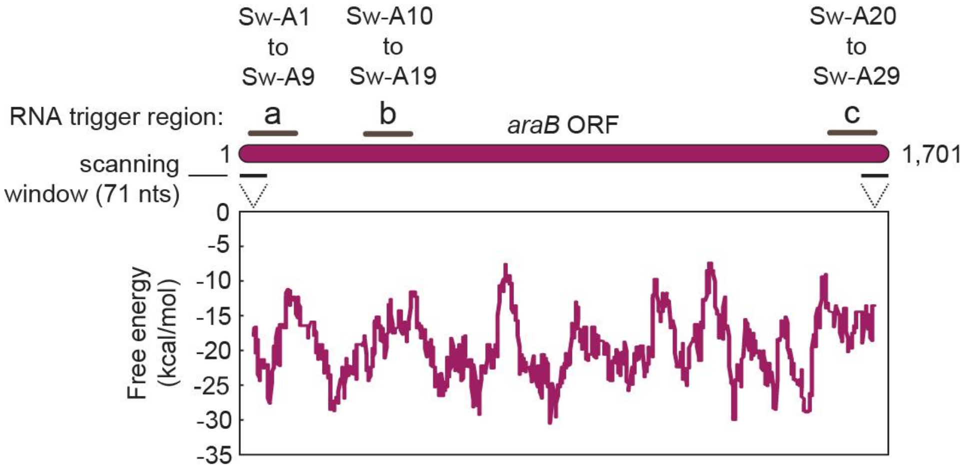

Figure S8. Regions within the *araB* open-reading frame selected for RNA triggers based on local secondary structure. The 1701-nt ORF was scanned using a 71-nt window to calculate the predicted folding free energy at each position. Three regions were selected based on their proximity to the 5′ end (a) or weaker free energies (b, c). A total of 29 gRNA switches were then designed (Sw-A1 - Sw-A29), with RNA triggers derived from the three regions.

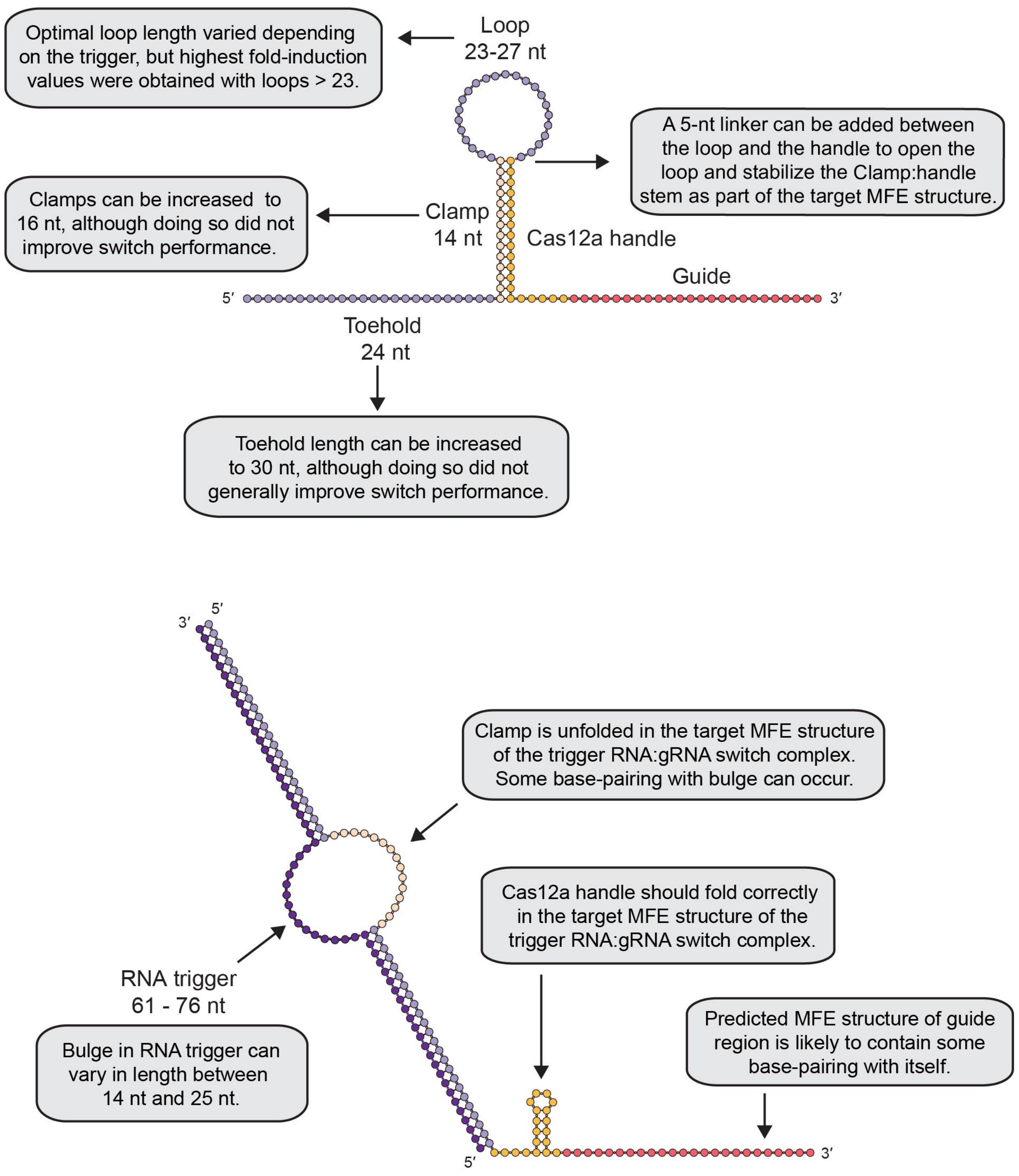

Figure S9. Guidelines for designing Cas12a gRNA switches. The guidelines are based on the experimental results obtained through this work.

SUPPLEMENTARY TEXT

Text S1. NUPACK code for designing an artificially triggered CRISPR gRNA switch with a 15-nt toehold, 13-nt clamp and 15-nt loop.

```
# FnCas12a CRISPR gRNA switch design
# Toehold: 15 Clamp: 13 Loop: 15 architecture. See NUPACK User Guide for valid options
material = rna
temperature = 37.0
trials = 10

### Structures: Target structures are : CRISPR switch alone, switch in complex
structure switch_off =  U15 D13 U15 U5 U1 U24
structure switch_on = D15 (U13 D15 + U13) U4 D5 U4 U1 U24

# Add domains here (Replace Ns of 'trig' domain for targeting natural RNA, and guide
sequence with desired CRISPR target)
domain trig = N43
domain toehold = N15
domain clamp = N13
domain loop = N15
domain handle = AATTTCTACTGTTGTAGAT #This is the processed FnCas12a handle
domain guide = N24 # Replace this with CRISPR target guide sequence

## Define strands
strand switchstrand = toehold clamp loop handle guide
strand triggerstrand = trig
switch_on.seq = trig toehold clamp loop handle guide
switch_off.seq = toehold clamp loop handle guide

## Other options
switch_on.stop = 5.0
prevent = AAAA, CCCC, GGGG, UUUU, KKKKKK, MMMMMM, RRRRRR, SSSSSS, WWWWWW, YYYYYY
```

Text S2. NUPACK code for designing Sw-R11, a natural triggered CRISPR gRNA switch with a 24-nt toehold, 14-nt clamp, 19-nt loop, and 5-nt linker.

```
# FnCas12a CRISPR gRNA switch design
# Toehold: 24 Clamp: 14 Loop: 19 Linker: 5 architecture. See NUPACK User Guide for
valid options
material = rna
temperature = 37.0
trials = 10

### Structures: Target structures are : CRISPR switch alone, switch in complex
structure switch_off =  U24 D14 (U19 U5) U4 U1 U24
structure switch_on = D19 (U25 D24 + U14) U5 U4 D5 U4 U1 U24

# Add domains here (Replace Ns of 'trig' domain for targeting natural RNA, and guide
sequence with desired CRISPR target)
domain trig = gcgatcaggaagaccctcgcggagaacctgaaagcacgacattgctcacattgcttccagtattactt
#This is RyhB, replace with other trigger if desired
domain toehold = N24
domain clamp = N14
domain loop = N19
domain linker = N5 # The linker optimises desired structures, it is the only sequence
that nupack will actually design as toehold and loop are fixed when using a fixed
trigger
domain handle = AATTTCTACTGTTGTAGAT #This is the processed FnCas12a handle
domain guide = CCTCTGGCGGTGATAATGGTTGCA #This is G1 guide, replace with Cas12a DNA
target

## Define strands
strand switchstrand = toehold clamp loop linker handle guide
strand triggerstrand = trig
switch_on.seq = trig toehold clamp loop linker handle guide
switch_off.seq = toehold clamp loop linker handle guide

#### Other options
switch_on.stop = 5.0
prevent = AAAA, CCCC, GGGG, UUUU, KKKKKK, MMMMMM, RRRRRR, SSSSSS, WWWWWW, YYYYYY
```

SUPPLEMENTARY REFERENCES

1. Zhang,X., Wang,J., Cheng,Q., Zheng,X., Zhao,G. and Wang,J. (2017) Multiplex gene regulation by CRISPR-ddCpf1. *Cell Discov*, **3**, 17018.
